# Design modification and optimisation of the perfusion system of a tri-axial bioreactor for tissue engineering

**DOI:** 10.1007/s00449-015-1371-1

**Published:** 2015-02-10

**Authors:** Husnah Hussein, David J. Williams, Yang Liu

**Affiliations:** Centre for Biological Engineering, The Wolfson School of Mechanical and Manufacturing Engineering, Loughborough University, Loughborough, LE11 3TU UK

**Keywords:** Bioreactor, Perfusion, Pressure, Tissue engineering

## Abstract

A systematic design of experiments (DOE) approach was used to optimize the perfusion process of a tri-axial bioreactor designed for translational tissue engineering exploiting mechanical stimuli and mechanotransduction. Four controllable design parameters affecting the perfusion process were identified in a cause–effect diagram as potential improvement opportunities. A screening process was used to separate out the factors that have the largest impact from the insignificant ones. DOE was employed to find the settings of the platen design, return tubing configuration and the elevation difference that minimise the load on the pump and variation in the perfusion process and improve the controllability of the perfusion pressures within the prescribed limits. DOE was very effective for gaining increased knowledge of the perfusion process and optimizing the process for improved functionality. It is hypothesized that the optimized perfusion system will result in improved biological performance and consistency.

## Introduction

The application of mechanical stimulation conditions that mimic the in vivo loading environment of a tissue can enhance cell metabolism, improve extracellular matrix synthesis, and improve the load bearing capacity of tissue engineered constructs [1–3]. In collaboration with the Healthcare Engineering group at Loughborough University, a combined compression, perfusion and pressurisation culture system was designed and built by Bose ElectroForce in response to the need of a bioreactor system that simultaneously applies loading conditions to mimic the complex loading environment that nucleus pulposus—a cartilage-like tissue experiences in vivo [4]. The concept of the bioreactor consists of a disc-shaped specimen, which is mounted between two porous platens and enclosed in a semi-permeable membrane sheath and subjected to axial compressive loads and radial hydrostatic pressure. The porous platens also allow perfusion of culture medium to the specimens to improve nutrient delivery to the cells. The system accommodates four samples each with an independent channel for nutrient supply and pressure control.

The initial design of the system was optimised for high pressure 50–300 kPa achieved by applying high flow rates of 5–50 ml/min. To further adapt such system to the low flow rates (0.5 ml/min or less) typically used for chondrogenesis [2, 5–7], it is necessary to modify some of the design and settings of the current system and optimise its functionality in terms of uniformity in the fluid flow environment and the ability to regulate pressures at low flow rate.

Flow variability can lead to undesirable differences in cellular growth rates on the three dimensional scaffolds due to differences in mass transport of oxygen, growth factors and other nutrients to the cells and the removal of waste products away from them during culture. This makes it difficult to compare results both within and between experiments.

Design of experiments was used as a problem solving quality tool to model and optimise the performance of various types of bioreactors and bioprocesses [8–10]. Here, such tools were also applied to optimise the tri-axial bioreactor perfusion performance at low flow rates, reduce the load on the pump and to gain a better understanding of the perfusion process. The paper is organized as follows: “The perfusion system of the tri-axial bioreactor” introduces the perfusion system of the tri-axial bioreactor. “Methodology for design of experiments” presents the methodologies that have been used for the designing purposes. “Experiment procedure” describes the procedure used for the experiment. “Determination of the optimal parameter settings that minimise the load on the pump and process variability” and “Determination of the root cause(s) of pressure instability” present the results and discussions and finally “Conclusions” concludes the experiment.

## The perfusion system of the tri-axial bioreactor

**Fig. 1:**
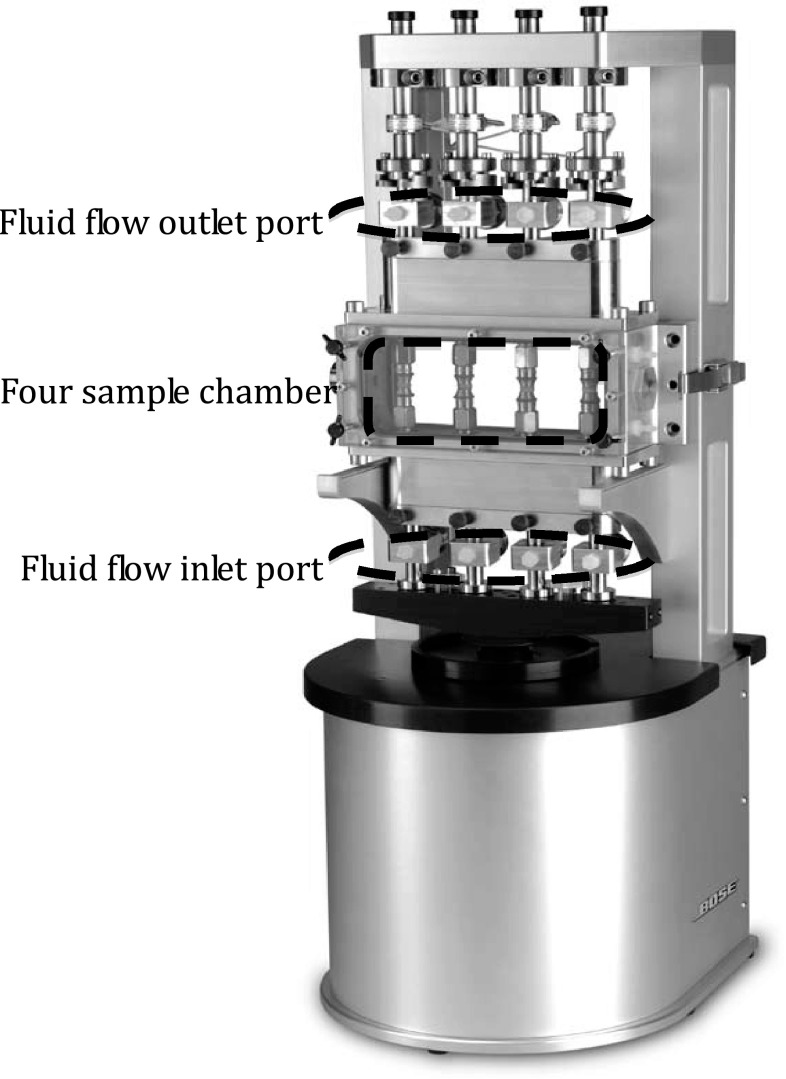
Loading frame of the tri-axial bioreactor system

The loading frame of the tri-axial bioreactor system is shown in Fig. 1. The four-sample chamber, fluid flow inlet ports and the outlet ports are indicated on the figure. The flow loop can be divided into two main segments: the inlet tubing segment that passes through the pump head of a four-channel peristaltic pump and circulates fluid from a reservoir bottle to the samples through the inlet ports; and the return tubing segment that connects to the outlet ports and returns fluid back to the reservoir to form a closed-loop flow system. A distributing manifold distributes a single flow stream from the reservoir into four parallel streams and a collecting manifold collects the streams into one discharge stream back to the reservoir. Tube restriction using pinch valves on the return flow tubes provides control of inlet flow pressures after the system is filled. Flow pressures are measured by eight pressure transducers that are connected on the bioreactor frame at the back of the flow inlet and outlet ports.

## Methodology for design of experiments

**Fig. 2:**
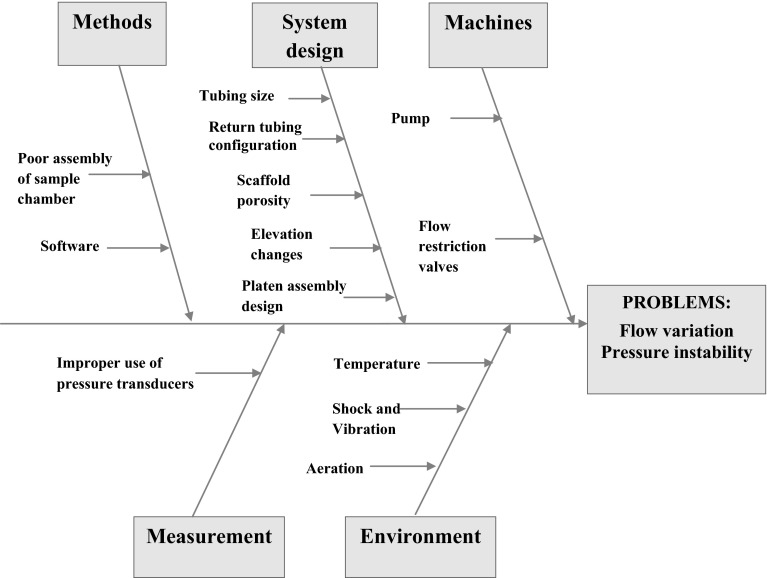
Cause-and-effect analysis of the problems

Firstly, a cause-and-effect diagram was used to identify potential experimental issues. Issues can be classified into six categories in a cause-and-effect diagram: environment, machines, materials, maintenance, measurement and methods [11, 12]. Based on scientific understanding or prior knowledge of the perfusion process, the cause-and-effect diagram was constructed as shown in Fig. 2. In the diagram, the process and design parameters which can influence the perfusion pressures are identified. The factors are grouped into five different categories that suit the tri-axial bioreactor situation: methods, system design, machines, measurement and environment.

Four controllable design parameters were identified in the cause-effect diagram as potential improvement opportunities: elevation changes, platen design, length of the inlet tubing segment and the configuration of the return tubing segment. A 2 level full factorial DOE experiment including replications would have required 32 runs. To reduce the number of experimental runs, the first step was to conduct a screening experiment to separate out the factors that have the largest impact from the insignificant ones. For instance, the length of the inlet tubing segment was found to have no effect on the pressure responses based on previous study. This is simply because there is no pressure measurement system within this section. Even if pressure transducers were connected, the tubing and fittings would only have a minimal effect due to the low flow rate used in the study.

A full factorial DOE was conducted to find the settings of the design parameters that optimise the perfusion process. Based on the results of the screening experiment (not shown), three factors were selected for the DOE: platen design (A), the configuration of the return tubing segments between the outlet ports of the bioreactor and the collecting manifold (B) and the elevation difference between the fluid surface level in the reservoir and the outlet ports of the bioreactor (C). Each design parameter was kept at two levels, which are shown in Table 1. Statistical experimental design and analysis was performed in Minitab software.

**Table 1:**
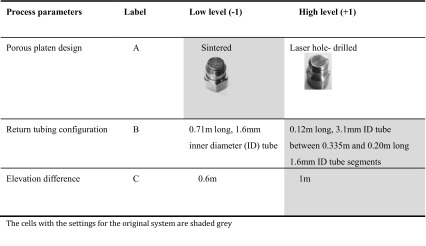
List of design parameters and their ranges for the DOE

## Experiment procedure

Before the inlet and return tubing segments were attached to the bioreactor frame, the sections were connected together using the male and female quick disconnect fittings attached at the end of the tubes and filled with water. The pump was then put on standby and the tubing segments were connected to their respective ports on the bioreactor frame. Once the complete system was assembled, the pump was restarted to fill it up. The system was drained in preparation for the next DOE run.

The filling up procedure described above was repeated for a second DOE followed by the application of manual pinch valves on the return flow segments between the outlet ports of the bioreactor and the collecting manifold to control the bioreactor inlet pressures between prescribed limits of 4–6 kPa. All experiments were conducted at a mass flow rate of 0.22 ml/min at a room temperature of 20 °C.

## Determination of the optimal parameter settings that minimise the load on the pump and process variability

Fluid flows through multiple changes in elevation between the inlet ports of the bioreactor and the end flow point at the reservoir. As the fluid rises through the bioreactor frame, the pump increases the fluid pressure to overcome the elevation increase and frictional forces in the bioreactor frame and to clear the fluid in the pre-filled return flow segment. The fluid loses its elevation energy as it propagates through the return flow segment to the reservoir.

**Fig. 3:**
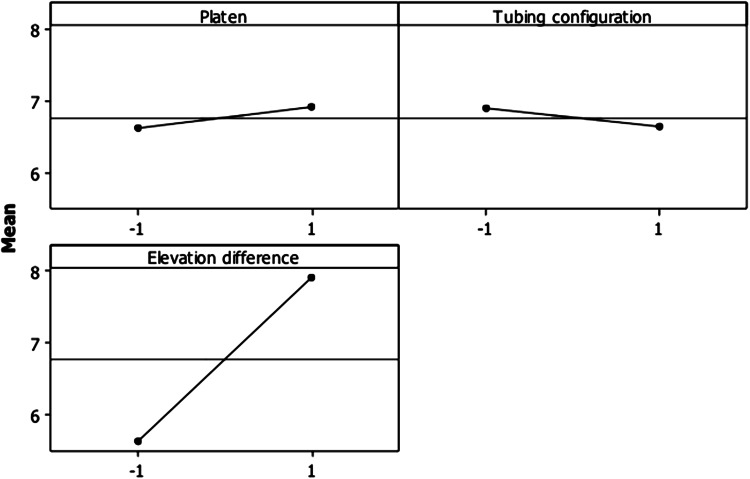
Main effects plot for the pressure increase supplied by the pump to displace the fluid in the pre-filled return flow segments and move fluid between the inlet and outlet ports of the bioreactor

The design parameter settings which minimise the load on the pump are the levels of the significant parameters which minimise the pressure increase during fluid elevation between the inlet and outlet ports of the bioreactor. This information was derived from the main effects plot shown in Fig. 3. The pressure increase is minimised when the elevation difference (C) is kept at a low level (−1), i.e. 0.6 m.

**Fig. 4:**
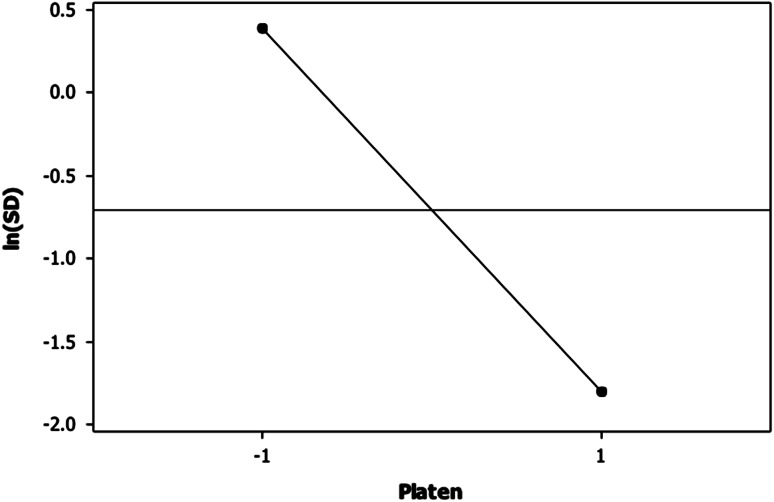
Main effects plot for the natural logarithm of the standard deviation ln(SD) of pressure drop between the inlet and outlet ports of the bioreactor

The best factor settings that minimise the variability in the process are the levels of the significant factors that minimise the variability in the pressure drop between the inlet and outlet ports of the bioreactor. Only the platen design (A) had a significant influence on the variation in the pressure drop. Analysis of factor A in the main effects plot (Fig. 4) showed that variation is maximised when the sintered platens (-1) are used in the sample chamber. This is because the sintered platens have non-uniform porous structures. Fluids always tend to follow the path of least resistance [13], which leads to preferential flow paths through the platens. Some regions in the platen were perfused, and others were left un-perfused. Another drawback with some sintered platen assemblies is that, most of the fluid can escape through small gaps between the porous platens and the sleeves in which the platens are mounted leaving the majority of the porous structures un-hydrated. This is due to the high flow restriction through the platen structures. In contrast, the laser hole-drilled platens (1) have uniform porous designs with low flow resistance. The porous structures were welded into the sleeves, thereby confining fluid flow to the porous platens. The laser hole-drilled design is the optimum platen design as it minimises variability in the pressure drop between the inlet and outlet ports of the bioreactor.

Since the return tubing configuration (B) does not appear to influence either the pressure increase or process variability, it is set at its economic level.

At the high level (1) of B, the larger diameter tubing connected between the two identical smaller diameter tubes costs more and takes up more culture medium. Therefore, it is more economical to use one 1.6 mm ID tubing segment between the outlets of the bioreactor and the collecting manifold. There is no trade-off in the selection of the factor levels to minimise the load on the pump and variability in the perfusion process. Therefore, the final optimum condition is given by:

- Platen design (A): high level (laser hole-drilled platens)
- Return tubing configuration (B): low level (1.6 mm ID tubing)
- Elevation difference (C): low level (0.6 m)

## Determination of the root cause(s) of pressure instability

Pressure instability is characterised by the slow increase of pressure, which can eventually exceed the hydrostatic pressure rating specification of the system leading to leakages. Usually, overpressure leakages first occur around the threaded connections to the inlet and outlet ports of the bioreactor frame. Further increase in pressure causes a leakage through the membrane that encloses the over-pressurised sample until there is no more fluid in the reservoir.

**Fig. 5:**
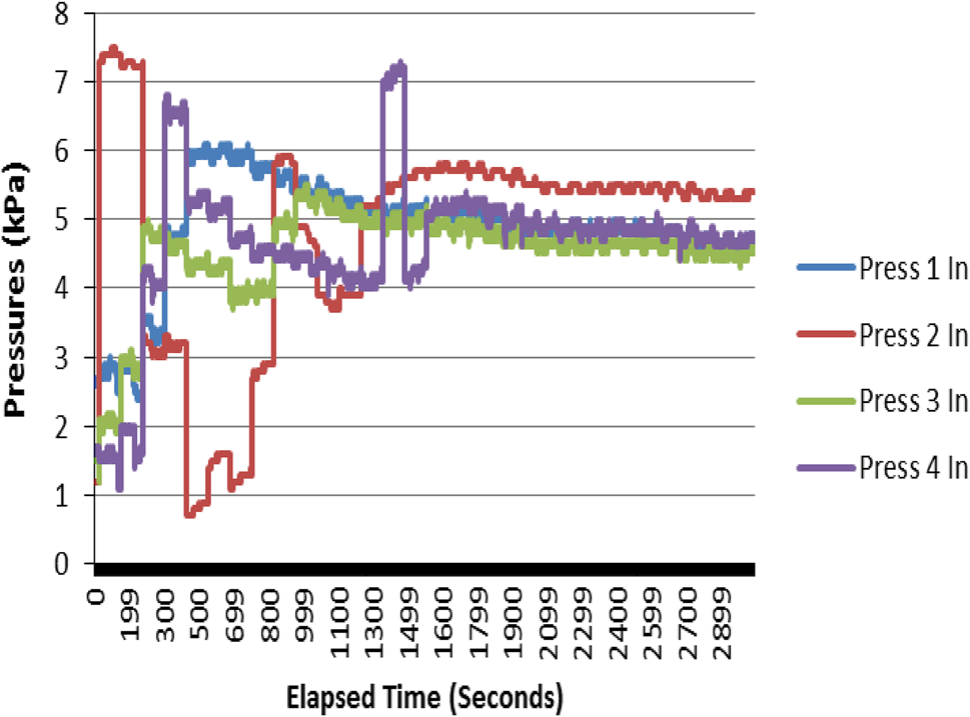
Pressure profiles at the beginning of the follow-up experiment

**Fig. 6:**
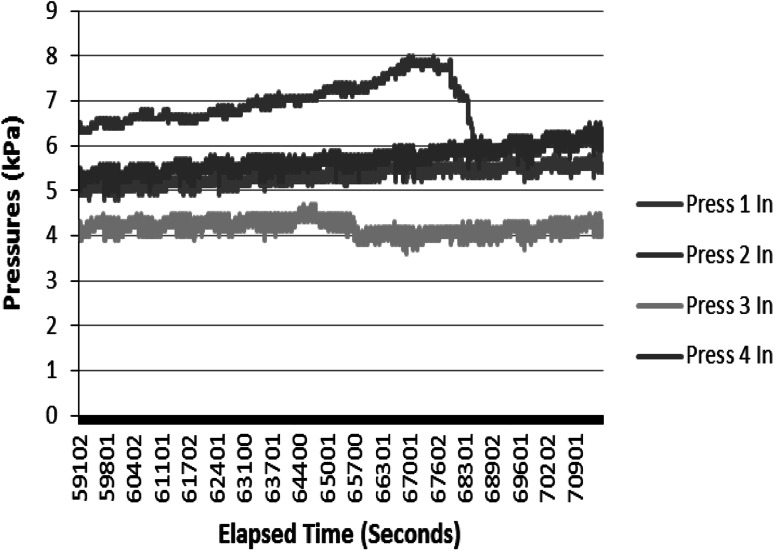
Pressure profiles during the last hours of the follow-up experiment

In preliminary experiments, pressure instability was observed with earlier experimental setups. Pressure responses were stabilised at the optimum condition that minimises the load on the pump and the variation in the perfusion process. This setup was selected for further analysis in a follow-up experiment conducted over a 19 h period. Pressures were initially stable within the prescribed limits after tweaking the pinch valves (Fig. 5). However, overnight, an upset condition of the system caused ‘Press 2 In’ (Fig. 6) to rise well beyond the prescribed limits. However, it appears that the cause of the increased pressure was cleared and the system remained stable until the operator opened the pinch valves the next morning.

The results of the follow-up experiment show that pressure instability is caused by noise factor(s), including trapped air pockets, causing blockages at low flow rates [14], especially when an air pocket is large enough to fill the diameter of the tubing or fitting, [15]. During this condition, the pressure of the liquid before the blockage rises and could dispel the air pocket if it is sufficient. The release of the air provides a pressure relief. However, if the pressure required to dispel the air pocket is beyond what the system pressure rating specification, then flow leakages occur.

There are two potential sources of air in the tri-axial bioreactor perfusion system. The first source is entrapment of air during filling, either initially or when the reservoir is changed, or the flow system is drained. When the flow system is empty, it is actually filled with air. While the system is filling, air pockets may become trapped at areas of high elevation [14, 15] for example, at the outlet ports of the bioreactor. The second source of air comes from the release of dissolved air in the perfused liquid, due to changes in pressure [16]. Experiment results have shown that the hydraulic fluid pumped through the tri-axial bioreactor contains between 5.5 and 8 % by volume of dissolved O_2_ and between 0.5 and 3.1 % of dissolved CO_2_. The air can come out of solution usually as a result of the sudden pressure drop as the fluid propagates downwards through the return flow segments during the filling up process and due to sudden release of the control valves. The air pockets tend to accumulate in the flow lines connecting to the bioreactor outlets and the collecting manifold and in the manifolds. The finding from the current research has illustrated the power of DOE as a troubleshooting quality tool. In all of the DOE runs, air pockets trapped in the return flow lines were dispelled by flicking them before the pinch valves were applied. The air pockets trapped in the manifolds could not be removed but did not cause overpressure issues.

**Fig. 7:**
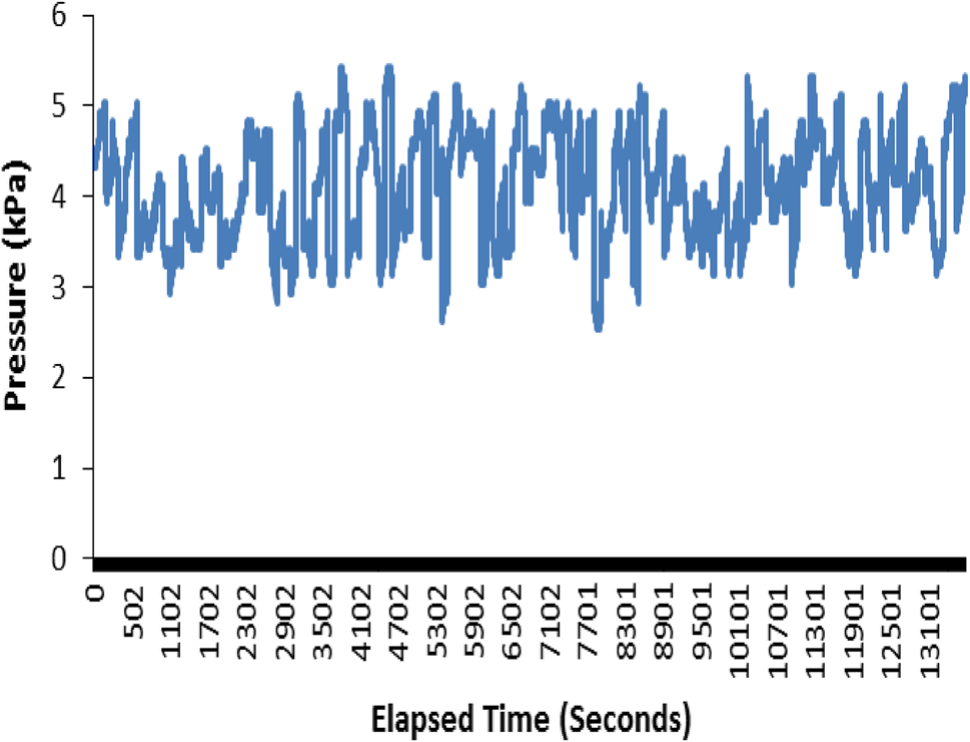
Pressure response using automated valves

Manual pressure control is disadvantageous as it requires the involvement of an operator to monitor the pressure transducers and tweak the valves when the pressures fall or rise above the target pressures. An automatic control system is needed to improve efficiency and stability. Consequently automated valves were supplied by Bose ElectroForce as part of the system and were fully integrated into the system’s hardware and software. The valves produce a repeatable pulsating pressure response as shown in Fig. 7. The system has been operated continuously for 5 days without any pressure control problems. In brief, this initial study has had a primary focus on the main effects. Improving the understanding of these main effects allows better control of the system and improves its reliability, such systems are very complex, allowing investigation of interactions in further work.

## Conclusions

With a modest amount of effort, we have gained a better understanding of the perfusion process, improved operational procedures and reduced variation in the process. Furthermore, cost savings have been made by removing unnecessary tubing lengths, equipment and fittings. This in turn has reduced the culture medium required to fill up the system by 25 %.

The optimization method presented can be applied to any previously designed system to understand the factors that affect the process, solve problems and reduce operational costs. But although cost savings can be made and functionality improved by optimising existing systems, numerical optimization technology combined with pump system optimization software present the greatest opportunities for bioreactor perfusion systems and other fluid flow systems.

It is hypothesised that the improved functionality of the perfusion system will lead to improved biological performance and biological performance consistency.
